# How Dual-Energy Contrast-Enhanced Spectral Mammography Can Provide Useful Clinical Information About Prognostic Factors in Breast Cancer Patients: A Systematic Review of Literature

**DOI:** 10.3389/fonc.2022.859838

**Published:** 2022-07-22

**Authors:** Federica Vasselli, Alessandra Fabi, Francesca Romana Ferranti, Maddalena Barba, Claudio Botti, Antonello Vidiri, Silvia Tommasin

**Affiliations:** ^1^ Radiology and Diagnostic Imaging, Istituto di Ricovero e Cura a Carattere Scientifico (IRCCS) Regina Elena National Cancer Institute, Rome, Italy; ^2^ Precision Medicine in Breast Cancer Unit, Fondazione Policlinico Universitario A. Gemelli, Istituto di Ricovero e Cura a Carattere Scientifico (IRCCS), Rome, Italy; ^3^ Division of Medical Oncology 2, Istituto di Ricovero e Cura a Carattere Scientifico (IRCCS) Regina Elena National Cancer Institute, Rome, Italy; ^4^ Division of Breast Surgery, Istituto di Ricovero e Cura a Carattere Scientifico (IRCCS) Regina Elena National Cancer Institute, Rome, Italy; ^5^ Human Neuroscience Department, Sapienza University of Rome, Rome, Italy; ^6^ Neuroimmunology Unit, Istituto di Ricovero e Cura a Carattere Scientifico (IRCCS) Fondazione Santa Lucia, Rome, Italy

**Keywords:** breast cancer: mammography, contrast-enhanced spectral mammography, HER2, progesterone, estrogen, Ki67

## Abstract

**Introduction:**

In the past decade, a new technique derived from full-field digital mammography has been developed, named contrast-enhanced spectral mammography (CESM). The aim of this study was to define the association between CESM findings and usual prognostic factors, such as estrogen receptors, progesterone receptors, HER2, and Ki67, in order to offer an updated overview of the state of the art for the early differential diagnosis of breast cancer and following personalized treatments.

**Materials and Methods:**

According to the PRISMA guidelines, two electronic databases (PubMed and Scopus) were investigated, using the following keywords: breast cancer AND (CESM OR contrast enhanced spectral mammography OR contrast enhanced dual energy mammography) AND (receptors OR prognostic factors OR HER2 OR progesterone OR estrogen OR Ki67). The search was concluded in August 2021. No restriction was applied to publication dates.

**Results:**

We obtained 28 articles from the research in PubMed and 114 articles from Scopus. After the removal of six replicas that were counted only once, out of 136 articles, 37 articles were reviews. Eight articles alone have tackled the relation between CESM imaging and ER, PR, HER2, and Ki67. When comparing radiological characterization of the lesions obtained by either CESM or contrast-enhanced MRI, they have a similar association with the proliferation of tumoral cells, as expressed by Ki-67. In CESM-enhanced lesions, the expression was found to be 100% for ER and 77.4% for PR, while moderate or high HER2 positivity was found in lesions with non-mass enhancement and with mass closely associated with a non-mass enhancement component. Conversely, the non-enhancing breast cancer lesions were not associated with any prognostic factor, such as ER, PR, HER2, and Ki67, which may be associated with the probability of showing enhancement. Radiomics on CESM images has the potential for non-invasive characterization of potentially heterogeneous tumors with different hormone receptor status.

**Conclusions:**

CESM enhancement is associated with the proliferation of tumoral cells, as well as to the expression of estrogen and progesterone receptors. As CESM is a relatively young imaging technique, a few related works were found; this may be due to the “off-label” modality. In the next few years, the role of CESM in breast cancer diagnostics will be more thoroughly investigated.

## Introduction

Breast cancer is the first cause of death in the female population in western countries (1). Early diagnosis and treatment have led to an increase in survival rate and better clinical outcome of women affected by breast cancer. However, up to 50% of patients may experience the relapse. Therefore, early identification of women at high risk of recurrence or who may benefit from treatment adjuvant setting is needed (2). Prognostic factors are essential to estimating individual patient risk of developing clinically silent micro-metastatic diseases and to determining patient eligibility for postsurgical systemic adjuvant therapy (3). The immunohistochemical prognostic factors that are assessed in order to plan a surgical and medical treatment for breast cancer are estrogen receptors (ER), progesterone receptors (PR), and epidermal growth factor (HER-2) (4). These factors, assessed on biopsy or surgical specimens, have permitted a classification in subtypes of breast cancer and a fine personalization of the treatment, thus tailoring the treatment in single cases. In addition to the abovementioned factors, also nuclear protein Ki-67 may influence the prognosis of the disease (5). Lastly, the histological grade is assessed in the diagnostic process (6) and used in the prognosis evaluation.

In mammography, breast cancer may not be identified due to the low difference between tumoral and background tissue x-ray attenuation (7), and to overcome this limit, during the past years, several studies have aimed at providing aid to physicians in the imaging analysis process, resulting in automated software able to improve sensitivity and specificity of diagnostic performances (8–10). Moreover, artificial intelligence (AI) has been applied to mammography and other imaging methodologies in cancer diagnosis, characterization, prognosis, and prediction of therapy outcome (11).

A recent diagnostic tool, with an improved background subtraction procedure, is the contrast-enhanced spectral mammography (CESM), a new technique derived from full-field digital mammography. CESM includes the administration of an iodine-based contrast material and the performance of low- (28–32 kV) and high-energy (45–49 kV) consecutive exposures to reveal areas of increased blood supply within the breast. In post-processing, these exposures are mutually subtracted in order to create a contrast-enhanced image and detect tumor vascularity (7). An image is acquired before contrast injection, and two more images are acquired about 2 min after contrast injection, one at low and the other at high energy. Postinjection images are combined in a single image that minimizes the appearance of breast tissue and increases the signal of an iodinated contrast agent (enhancement) (12). Recently, CESM has been becoming a valuable tool in the diagnosis and staging of primary breast cancer. It improves the diagnostic accuracy of mammography, providing a more accurate tumor sizing and the identification of multifocal diseases (13). Indeed, CESM improves the sensitivity for breast cancer detection without decreasing specificity, since it provides higher contrast and better lesion delineation as well as a better evaluation of lesion size and detects more multifocal breast cancers, than mammography alone or combined with ultrasonography (14–17). Similarly to breast magnetic resonance imaging (MRI), which is considered the gold standard in the assessment of tumor, the findings obtained with CESM examination suggest that it should be considered a useful tool in the evaluation of disease extension. As a matter of fact, both CESM and MRI may also evaluate tumor response during neoadjuvant chemotherapy (NAC), which, reducing tumor volume and metastasis occurrence, increases the probability of a positive response to breast-conserving surgery, to be used instead of mastectomy, and of a high survival rate in advanced breast cancer (18).

The aim of this study was to define the association between CESM findings and prognostic factors, such as ER, PR, HER2, and Ki67, with the aim to offer an updated overview of state of the art for the early differential diagnosis of breast cancer and the following personalized treatments. In this framework, we performed a systematic review of the literature.

## Materials and Methods

According to the PRISMA guidelines (19), two electronic databases (PubMed and Scopus) were used to perform the literature investigation, using the following keywords: breast cancer AND (CESM OR contrast enhanced spectral mammography OR contrast enhanced dual energy mammography) AND (receptors OR prognostic factors OR her2 OR progesterone OR estrogen OR Ki67). The search was concluded in August 2021. No restriction was applied to publication dates.

**Figure 1:**
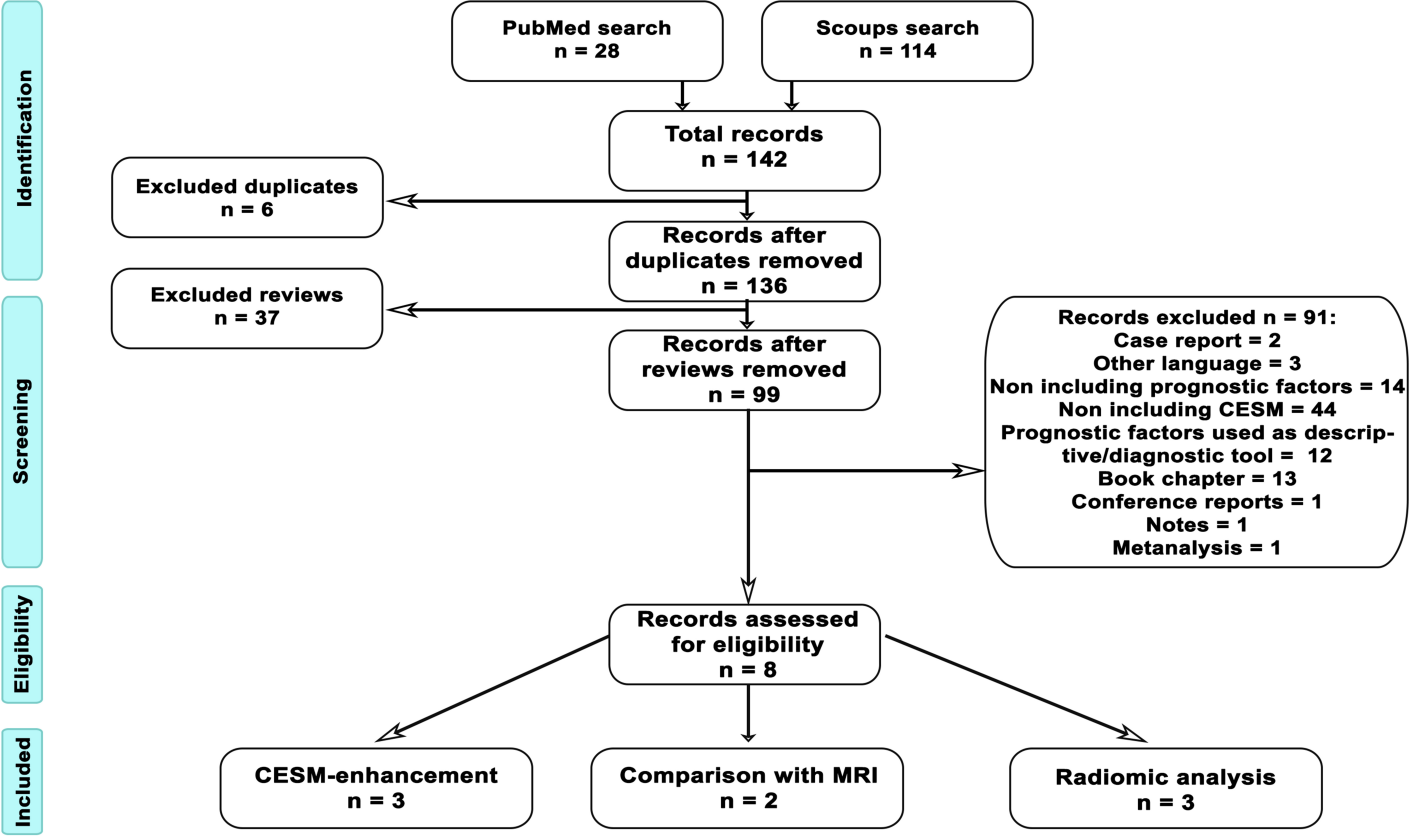
Flowchart of article selection. The procedure to identify suitable articles to be included in the systematic review was performed following PRISMA guidelines and schematized in the figure. From database search, we identified 28 articles in PubMed and 114 in Scopus, with a total of 142 records. Six of the retrieved articles were duplicated across the two databases; therefore, we further investigated 136 articles. Out of those, 37 articles were reviews and, after abstract examination, 99 more articl es were excluded, because they were case reports (2), were written in no English language (3), did not include prognostic factors (14), did not include CESM (44), used prognostic factors as diagnostic characterization (12), were book chapters (13), were conference reports (1), were notes (1), and were meta-analyses (1). Finally, eight records were assessed to be eligible. Specifically, three articles addressed CESM enhancement, two articles compared CESM and MRI, and three articles investigated radiomic analysis of CESM images.

First, we identified all documents in both databases. After identifying existing studies, we cross-checked all the collected articles to avoid duplicates. Abstracts were examined carefully, and the following exclusion criteria were applied: not a research article (e.g., review, book chapter, conference report, case report, meta-analysis), articles written in languages other than English, and articles investigating diagnostic methodologies other than CESM or not investigating prognostic factors. The flowchart of article selection is shown in **Figure 1**.

To assess the scientific quality of the studies included in our review and any possible source of bias, we prepared a checklist of questions in accordance with QUADAS guidelines (20). The overall procedure was carried out by two investigators (FV, ST).

## Results

We obtained 28 articles from the research in PubMed and 114 articles from Scopus. After the removal of six replicas that were counted only once, out of 136 articles, 37 articles were reviews and were removed. The abstracts of the remaining 99 articles were inspected to verify conformity to exclusion criteria: 2 articles were case reports, 3 were written in a non-English language, 14 did not include prognostic factors, 44 did not include CESM, 12 used prognostic factors as diagnostic characterization, 13 were book chapters, 1 was a conference report, 1 was a note, and 1 was a meta-analysis. Finally, eight articles tackled the relation between CESM imaging and ER, PR, HER2, and Ki67. Among the eight articles admissible for the following analysis, the relation between CESM and prognostic factors was investigated with CESM-MRI comparison (two articles), with CESM enhancement (three articles), and with radiomic analysis of CESM enhancement (three articles).

Since CESM is a recent diagnostic technique, articles investigating how CESM may provide clinical information on biological prognostic factors date back to the last 2 years.

### CESM MRI Comparison

CESM is often compared to MRI to test its utility in tumor diagnosis, and indeed, enhancement patterns were moderately in agreement between the two techniques (21). CESM may produce an enhancement intensity weaker in the ER-positive group than in the ER-negative group, as well as weaker in the PR-positive group than in the PR-negative group, and stronger in the HER-2-positive group than in the HER-2-negative group (21). Further, when comparing radiological characterization of the lesions obtained by either CESM or contrast-enhanced MRI, they have a similar association with the proliferation of tumoral cells, as expressed by Ki-67 (22). However, the authors do not describe if there are any differences between CESM and MRI in differentiating hormonal receptor status.

### CESM Enhancement

In CESM-enhanced lesions, the expression was found to be 100% for ER and 77.4% for PR, while moderate or high HER2 positivity was found in lesions with non-mass enhancement and with mass closely associated with a non-mass enhancement component (23). Further, *via* CESM enhancement, neoplasms larger than 5 mm, with a high proliferative index and frequently HER2-positive, are recognized (24). Conversely, the non-enhancing breast cancer lesions were not associated with any prognostic factor, such as status of ER and/or PR, HER2 expression and/or amplification, and percentage of Ki67, which might be associated with the probability of showing enhancement (25).

### Radiomic Analysis

Nowadays, one of the cutting-edge methods for image analysis is based on radiomics. For non-invasively assessing the hormone receptor status, other than tumor invasiveness and grade, radiomic features were derived from the first-order histogram of primary breast cancer lesions contoured on both CESM and MRI images and the two techniques resulted to be alternative in the assessment of hormone receptor status (26). Further, radiomics on CESM images showed the potential for the non-invasive characterization of heterogeneous tumors with different hormone receptor statuses (27). Lastly, radiomic features may predict histological outcomes and molecular subtypes *via* discriminating lesions with a positive or negative expression of hormonal receptors, and being associated with HER2. In particular, in an immunohistochemical study, the performances for discriminating positive versus negative expressions were 90.87% for HER2 positive versus HER2 negative, 83.79% for ER positive versus ER negative, and 84.80% for Ki67 positive versus Ki67 negative (28). The list of the final articles and their relationship with biologic prognostic factors is summarized in **Table 1**.

**Table 1:** Characteristics of articles investigating the relationship between dual-energy contrast enhanced spectral mammography (CESM) and biologic prognostic factors.

Study	Participants	Standard references	Hormonal prognostic factor	Analysis technique	Radiological feature	Results
(23)	31 women (mean age 57.1 years; range 41–78)	Definitive histology	ER PR HER2 Ki67	CESM-histology agreement in lesion size measurement	Focus, ME and NME, and diameter	The totality of the lesions had a receptor positivity to estrogens. NME is associated with HER2 positivity:
(25)	348 women (mean age 60.1 years; 11.93 years; range 37–88)	Definitive histology	ER PR HER2 Ki67	CESM enhancement at lesion site	CESM enhancement	HER2 negative molecular subtype associated with higher probability of enhancement. False negative lesions are not associated with hormonal status
(24)	34 women (median age 53.9 years, 8.5 years)	Definitive histology	ER PR HER2 Ki67	Manually contoured lesions	CESM enhancement at calcification site	Association between enhancement and expression of Ki-67, HER-2; ER, PG
(28)	52 women (median age 50 years; 1st quartile 45.75, 3rd 60.25 years; range 37–80)	Diagnostic biopsy	ER PR HER2 Ki67	Radiomics of manually outlined ROIs	Mean, VC, difference between max and min gray level, SK, EN, RS and kurtosis.	Multivariate analysis of the histogram features can discriminate lesions with positive ER, PG, and Ki67 from lesions with negative ER, PG, and Ki67
(21)	131 women (mean age 42 years; range 18–77)	Diagnostic biopsy or definitive histology	ER PR HER2	CNR and relative signal difference	CESM enhancements	Enhancement of ER positive lesions < ER negative lesions. Enhancement of PR positive lesions < PR negative lesions. Enhancement of HER 2 positive lesions > HER2 negative lesions.
(26)	48 women (mean age 50.7 ± 8 years; range 38–74)	Diagnostic biopsy	ER PR HER2	Radiomics of manually contoured lesions	COM, RLM, GRA, ARM, WAV, GEO.	HR positivity and HR negativity differentiation accuracy observed.
(27)	100 women (mean age 51.5 years; 12 years; range 25-79)	Definitive histology	ER PR HER2	Radiomics of manually contoured lesions	HIS, COM, RLM, WAV.	HR positivity and HR negativity differentiation accuracy. HER2 positivity/HR negativity and HER2 negativity/HR positivity differentiation accuracy. Triple-negative and triple-positive differentiation accuracy.
(22)	100 women (range 42–80; median 58; 10.2)	Diagnostic biopsy for benign lesions Definitive histology for malignant lesions	ER PR HER2 Ki6	CESM enhancement	BI-RADS classification	Ki-67 correlation with CESM BIRADS.

ER, estrogen receptors; PR, progesterone receptors; HER2, epidermal growth factor; CESM, contrast-enhanced spectral mammography; ME, mass enhancement; NME, non-mass enhancement; ROI, region of interest; CNR, contrast noise ratio; COM, co-occurrence matrix; RLM, run-length matrix; GRA, absolute gradient; ARM, autoregressive model; WAV, discrete Haar wavelet transform; GEO, lesion geometry; MI, mutual information; VC, variation coefficient; SK, skewness; EN, entropy; RS, relative smoothness; BI-RADS, Breast Imaging Report and Data System.

### Quality Assessment

The Quadas-2 survey showed that the articles considered in the analysis were at risk of bias, especially for what concerns the study tests conducted in each research. Indeed, at the time of the radiological evaluation the investigators, i.e., the radiologists, were aware of the results of the histological test, in all studies with the exception of one study, who performed a blinded histological analysis (22). However, all articles referred to a proper reference test, i.e., definitive histology or diagnostic biopsy. Further, four articles were biased in patient selection, because they removed either patients with a tumor not easily identifiable, such as that with suspicious but not contrast-enhancing lesions (27), or patients with post-histology edema or not willing to undergo CESM (25), or because they did not clarify whether patients have mono- or multifocal diseases (21, 22). Lastly, one article alone was at risk of flow bias, because of using both definitive histology and diagnostic biopsy as standard reference (21). The Quadas-2 survey is shown in **Table 2**.

**Table 2:** QUADAS2.

	Bias risk				Applicability issue		
	Patient selection	Study test	Standard reference	Timing and flow	Patient selection	Study test	Standard reference
(23)	YES	NO	YES	YES	YES	YES	YES
(25)	NO	NO	YES	YES	NO	YES	YES
(24)	YES	NO	YES	YES	YES	YES	YES
(26)	YES	NO	YES	YES	YES	YES	YES
(27)	NO	NO	YES	YES	YES	YES	YES
(21)	NO	NO	YES	NO	YES	YES	YES
(28)	YES	NO	YES	YES	YES	YES	YES
(22)	NO	YES	YES	YES	YES	YES	YES

Articles fulfilling (YES) or not fulfilling (NO) QUADAS2 criteria to assess the study quality.

## Discussion

CESM is a very young modality recently introduced in the breast imaging scenario; therefore, the eligible articles found on our research were not older than 2 years. CESM shows considerable promise as the primary imaging test in symptomatic patients, providing improved diagnostic and staging information at the first evaluation.

### Prognostic Factors

Prognostic factors are correlated with patient prognosis and allow important information about the efficacy of antitumoral treatment. Literature demonstrated that proliferative activity indicator (Ki67), HER-2, and hormonal receptor, such as ER and PR, statuses are important in treatment choice and that they have prognostic value in predicting pathological response and clinical outcome (29). As a matter of fact, HER-2 status represents a solid prognostic factor that predicts the response to trastuzumab alone or associated with pertuzumab treatment in locally advanced or early disease therapy (30). Also, determination of ER and PR status is crucial as their expression on the tumor cellular surface is related to a good response to endocrine therapy in both neoadjuvant and adjuvant therapy (30).

Among the biomarkers used to define tumor aggressiveness, Ki67, HER-2, ER, and PR are quantitative values. On the contrary, grading, which is used as well to define tumor aggressiveness, is a qualitative biomarker; therefore, we rather avoided to include it as an investigated prognostic factor in this study.

### Comparison Between CESM and MRI and the Association With Prognostic Factors

CESM is a recent tool for diagnostic imaging that, although it uses ionizing radiations thus presenting some limitations in terms of radioprotection (7), may overtake the use of MRI in breast cancer monitoring, since it is more accessible, cheaper, faster, and more tolerated by patients (31), while maintaining performance equivalent to MRI and improving specificity (7, 17). As a matter of fact, the promising results of diagnostic performance could suggest CESM to be a valid alternative for patients who are not eligible for MRI. As a matter of fact, CESM and breast MRI similarly detect physiological, benign background parenchymal enhancement, which may be significantly associated with menopausal status, radiation therapy, hormonal treatment, and breast density and that rarely causes diagnostic issues if showing a bilateral, symmetrical appearance (32, 33). At the same time, the background parenchymal enhancement on MRI is considered a biomarker for increased risk of breast malignancy, while it is not known if the same holds true for CESM (34, 35).

Indeed, CESM and MRI show similar enhancement patterns (21) and a similar association with the proliferation of tumoral cells (22). The equivalence of CESM and MRI might rise from tumor vascularization, which is a crucial feature observed by both diagnostic modalities and is influenced by Ki67.

A necessary step to include CESM in everyday clinical practice will be the standardization of diagnostic criteria. Given the similarity of the basic principles of lesion blood supply of the two modalities, MRI morphology descriptors have been already investigated and used to characterize lesions on CESM (36); however, more studies are needed to finalize the use of these descriptors in CESM image evaluation.

As in any imaging modality, patient motion may affect image quality. Due to the simultaneous acquisition of low-energy and high-energy images, the length of each exposure with CESM is longer than a standard full-field digital mammography, increasing the possibility of motion. However, the examination time of CESM is still shorter than the second-level examination MRI, reducing the risk of motion artifacts. Moreover, to instruct well the patient to hold as still as possible during the exposure is fundamental to reducing the possibility of motion (37).

### CESM Enhancement and Prognostic Factors

CESM combines an iodinated contrast agent with the standard mammographic technique to improve lesion detectability. Since the growth of tumors is accompanied by angiogenesis, CESM permits to assess the enhancement related to the neovascularity of breast cancers, allowing a functional characterization in addition to the morphological features provided by structural images (16).

In literature, CESM-enhancing lesions have been associated with higher levels of prognostic factors, such as ER, PR, and HER2 (23, 24). On the other hand, non-enhancing lesions have been found not to relate to prognostic factors (25). Indeed, tumors have a higher enhancement compared to normal tissue due to the increase in vascularization, which in turn is associated with different tumor characteristics and therefore different expressions of prognostic factors.

### CESM-Based Radiomic Analysis and Prognostic Factors

Feature extraction in radiomics is typically realized by means of pattern recognition algorithms and provides, as a result, a set of numbers, each one representing a quantitative description of a specific either geometric or physical property of the image portion under consideration. In oncological applications, examples of features are tumor size, shape, intensity, and texture, collectively providing a comprehensive tumor characterization, called the radiomics signature of the tumor (38). From an epistemological perspective, radiomics is based on the hypothesis that the extracted features reflect mechanisms occurring at genetic and molecular levels (39) and may reveal the relationship of tumor lesion surfaces with prognostic factor expression. The potential of radiomics applied on breast imaging has been investigated recently, and studies have already demonstrated the additive value of radiomics on MRI in breast cancer evaluation and prognosis (40, 41). Indeed, radiomics on CESM images might assess hormone receptor status (26) and characterize the related heterogeneous tumors (27), as well as predict histological outcomes and molecular subtypes associated with hormone receptors’ expression (28). As a matter of fact, radiomics arises from the analysis of cell morphology, which may be influenced by the expression of the different receptors on the cell surface of the different tumors, thus permitting to differentiate the receptor status starting from imaging.

Radiomics could also contribute to differentiating benign from malignant enhancement in complicated cases, as in patients with high background parenchymal enhancement or low vascularized lesions, that may have a high risk for underestimation or even overestimation of the lesion (42), and to predicting response to NAC (43).

### Literature

The articles included are all published in the last 2 years, and none of them was blinded, except that of Petrillo et al. (22). Nevertheless, this bias did not invalidate the articles, as the goal of these studies was to find a relationship between imaging features and prognostic factors, not just detecting a tumor. Indeed, knowing the histological subtypes was part of patients’ preliminary information needed to obtain a sample of patients with heterogeneous radiologic patterns. Conversely, homogeneous histologic analysis was crucial in order to obtain consistent results among patients, and in one article the authors did not grant this consistency.

Only eight articles investigated the association between CESM imaging and prognostic factors, suggesting that the use of this technique in cancer prognosis and monitoring is still to be deeply investigated. Indeed, the modality is relatively young and large data pools are required to get strong results on this topic.

## Conclusion

CESM is a relatively young diagnostic tool, and our review showed its potential on finding a precise imaging semeiotic, thanks to its association with prognostic factors, in order to provide patients with the most accurate pre-therapy and surgery evaluation. In this review, CESM enhancement showed an association with the proliferation of tumoral cells, as well as the expression of estrogen and progesterone receptors, although there is not a certain correlation between specific patterns of enhancement and prognostic factor outlines. Future studies might investigate CESM’s ability in identifying ER/PR positivity and HER2 positivity/amplification, as, so far, they have not been investigated. Moreover, even if recent studies have investigated the radiomic application on CESM (26, 28), more results are requested to enforce these promising applications.

As CESM is a relatively young imaging technique, literature shows a few related works, often suffering from bias risk, and this is certainly due to the “off-label” use in clinical practice. The role of CESM in breast cancer diagnostics will be further investigated, and radiomics studies will provide further predictive and prognostic information on the clinical impact of this technique.

## Author Contributions

Study concept and design, analysis and interpretation of data: FV and ST; statistical analysis: MB and CB; drafting of the manuscript: AF, FF, and AV; critical revision and final approval of the manuscript: all authors. All authors contributed to the article and approved the submitted version.

## Funding

This work was supported by Funds Ricerca Corrente 2022 from Italian Ministry of Health.

## Conflict of Interest

The authors declare that the research was conducted in the absence of any commercial or financial relationships that could be construed as a potential conflict of interest.

## Publisher’s Note

All claims expressed in this article are solely those of the authors and do not necessarily represent those of their affiliated organizations, or those of the publisher, the editors and the reviewers. Any product that may be evaluated in this article, or claim that may be made by its manufacturer, is not guaranteed or endorsed by the publisher.
